# Effect of apoA-I on cholesterol release and apoE secretion in human mature adipocytes

**DOI:** 10.1186/1476-511X-9-75

**Published:** 2010-07-20

**Authors:** Karima Bencharif, Laurence Hoareau, Ravi K Murumalla, Evelyne Tarnus, Frank Tallet, Roger G Clerc, Christophe Gardes, Maya Cesari, Régis Roche

**Affiliations:** 1 LBGM-GEICO, Laboratoire de Biochimie et de Génétique Moléculaire - Groupe d'Etude sur l'Inflammation Chronique et l'Obésité, Plateforme CYROI, Université de La Réunion 15 avenue René Cassin 97715 Saint Denis Messag Cedex 9, France; 2 Service de biochimie, Centre Hospitalier Félix Guyon, 97400 Saint Denis, Ile de La Réunion, France; 3 Metabolic Disease Therapeutic Area, F. Hoffmann-La Roche Ltd., 124 Grenzacherstrasse, 4070 Basel, Switzerland

## Abstract

**Background:**

The risk of cardiovascular disease is inversely correlated to level of plasma HDL-c. Moreover, reverse cholesterol transport (RCT) from peripheral tissues to the liver is the most widely accepted mechanism linked to the anti-atherosclerotic activity of HDL. The apolipoprotein A-I (apoA-I) and the ABC transporters play a key role in this process.

Adipose tissue constitutes the body's largest pool of free cholesterol. The adipose cell could therefore be regarded as a key factor in cholesterol homeostasis. The present study investigates the capacity of primary cultures of mature human adipocytes to release cholesterol and explores the relationships between apoA-I, ABCA1, and apoE as well as the signaling pathways that could be potentially involved.

**Results:**

We demonstrate that apoA-I induces a strong increase in cholesterol release and apoE secretion from adipocytes, whereas it has no transcriptional effect on ABCA1 or apoE genes. Furthermore, brefeldin A (BFA), an intracellular trafficking inhibitor, reduces basal cholesterol and apoE secretion, but does not modify induction by apoA-I. The use of statins also demonstrates that apoA-I stimulated cholesterol release is independent of HMG-CoA reductase activation.

**Conclusion:**

Our work highlights the fact that adipose tissue, and particularly adipocytes, may largely contribute to RCT *via *a mechanism specifically regulated within these cells. This further supports the argument that adipose tissue must be regarded as a major factor in the development of cardiovascular diseases, in particular atherosclerosis.

## Background

Epidemiological studies have repeatedly highlighted a strong inverse correlation between plasma concentrations of high-density lipoprotein cholesterol (HDL-c) and the risk of developing cardiovascular diseases, in particular atherosclerosis, in humans. Thus, low levels of plasma HDL-c increase the cardiovascular risk factor [1]. Reverse cholesterol transport (RCT) from peripheral tissues to the liver is a physiological process that enables the negative regulation of cholesterol deposits *via *the very low-density and low-density lipoprotein (VLDL and LDL). During reverse transport, high-density lipoproteins (HDL) take up cholesterol from peripheral cells and carry it to the liver. This process constitutes an initial and crucial step in cholesterol homeostasis in mammals, especially since cholesterol is cytotoxic when present in excess quantity [2]. Moreover, the concept of RCT from macrophages to the liver, and, ultimately to biliary excretion sytem, is the most documented mechanism to explain the ability of HDL to protect against atherosclerosis.

RCT from peripheral cells to the liver is a multi-step process. Initially, poorly lipidated apoA-I and small discoidal shaped particles, preß-HDL, take up cholesterol from peripheral cells. This results in a radical change, giving rise to spherical HDL3 then HDL2 particles, due to the fact that particles become enriched in esterified cholesterol (*via *lecithin cholesterol acyl transferase (LCAT) associated with preß-HDL particles) and phospholipids. The final uptake of HDL2 by the liver involves a selective receptor, the scavenger receptor B1 (SR-B1).

Apolipoprotein A-I (apoA-I), the major HDL apoprotein, plays a critical role in RCT. The binding of apoA-I to the ATP binding cassette transporter A1 (ABCA1) enables transmembrane transport of free cholesterol and phospholipids from peripheral cells into preß-HDL [3]. In addition, apoA-I also stimulates the secretion of apolipoprotein E (apoE); this action is probably partially dependent on ABCA1 [4]. However, it's well established that apoA-I, apoE, and ABCA1 closely cooperate to optimize the cellular mobility of cholesterol that leads to the formation of the HDLs [5]. ApoE plays a key role in this process, contributing both to the efflux of cholesterol [5], and to the expansion in the size of HDLs, by increasing the activity of LCAT, which esterifies free cholesterol within the HDL particles [6]. HDLs enriched in apoE (HDL1 or HDL-with apoE) are also taken up by the liver *via *the apoB/apoE LDL receptors (LDLR). In addition, Bernier *et al*, in collaboration with our laboratory, has demonstrated that overexpression of apoE in adipocytes reduces differentiation as well as the accumulation of cholesterol and triglycerides in these cells [7]. These findings partly explain the strong anti-atherogenic effect of this apoprotein.

The mechanisms involved in cholesterol efflux have already been described with fundamental differences related to the cell type studied. Indeed, it is known that cAMP is able to stimulate the efflux of cholesterol in murine macrophages and in RAW 264 cells [8,9], whereas this effect is not detectable in human THP-1 cells or in human monocyte derived macrophages [10,11]. Moreover, PKA activators (including cAMP) are known to stimulate efflux, whereas PKA inhibitors reduce efflux [12,13]. Finally, it is also known that PKC activators are capable of activating cholesterol efflux in fibroblasts, vascular smooth muscle cells, and in human macrophages [14,15], whereas inhibitors of PKC lead to a reduction in apoA-I induced efflux [16].

In addition, no data is currently available on the signalling pathways involved in the apoA-I induced secretion of apoE.

Significantly, the vast majority of studies related to RCT have used macrophage cell lines or primary cultures loaded with cholesterol *in vitro*. However, adipose tissue is also capable of spontaneously accumulating cholesterol and constitutes the most important storage tissue of cholesterol in the human body. Adipocytes may therefore be regarded as a "storage buffer", which may contribute to the regulation of plasma cholesterol. We have therefore aimed to investigate the first step of the RCT process, the cholesterol efflux, in primary cultures of mature human adipocytes (full fat), and to explore the relationships between apoA-I, ABCA1, and apoE along different signaling pathways that may potentially be involved in this process.

## Methods

### Materials

ApoA-I was purchased from Biomedical Technologies Inc. (Stoughton, USA). Adenylate cyclase inhibitor (ACi) was purchased from Calbiochem (USA). Rp-8-Br-cAMP, PKA inhibitor (PKAi), brefeldin A (BFA) and forskolin (FSK) were obtained from Clinisciences (Montrouge, France). Simvastatin was purchased from Cayman (USA).

### Origin of adipose tissue samples

Subcutaneous (abdominal, buttocks, hips and thighs) tissue samples of human white fat were obtained from normal weight or slightly overweight human subjects (exclusively females, mean body mass index 24.23) undergoing liposuction, performed under general anesthesia, for cosmetic reasons (aged between 25 and 60 years, mean 39 years). Apart from oral contraception, the subjects were not receiving treatment with prescribed medication at the time of liposuction. A total of 27 samples were obtained from 27 patients. The study was approved by the Ile de La Réunion ethics committee for the protection of persons undergoing biomedical research.

### Primary culture of human adipocytes

Cultures were prepared as previously described [17]. Briefly, tissue samples obtained by liposuction were digested for 30 min at 37°C in Ringer-Lactate buffer containing 1.5 mg/ml collagenase (NB5, SERVA, Germany, PZ activity 0.131 U/mg). The floating adipocytes (mature adipocytes) were rinsed three times in Ringer-Lactate. Cells were plated in 24-well (30 000 cells) or 6-well (120 000 cells) tissue culture plates with 199 culture medium supplemented with: 1% Fetal Bovine Serum (FBS) (PAN Biotech, France), amphotericin B, (5 μg/mL), streptomycin (0.2 mg/mL) & penicillin (200 U/mL) (PAN Biotech, France), 66 nM insulin (Umuline Rapide, Lilly, France), 2 g/L glucose, 8 μg/L biotin and 4 μg/mL pantothenate. Cells were then maintained at 37°C in 5% CO_2 _for a period of 24 hours prior to the experiments.

### Total cholesterol release

In the experiment to determine the apoA-I stimulated total cholesterol release, cells were treated with apoA-I (5, 10, 20 μg/ml) for 4, 6, 12 and 24 hours.

In inhibition experiments, mature adipocytes were pre-incubated for 1 hour with PKAi (40 nM), rp-8-Br-cAMP (40 μM) and ACi (20 μM). The medium was removed and replaced with medium containing inhibitors, with or without apoA-I (10 μg/ml) for 12 hours.

In the BFA and statin experiments, cells were pre-incubated for 12 hours with BFA (5 μg/ml) and for 1 hour with simvastatin (10 and 100 nM). Medium was removed and replaced with either medium alone or medium containing apoA-I (10 μg/ml) for 12 hours. BFA and simvastatin were present throughout this incubation time. Cell media were then collected and assayed for cholesterol content with a Cayman's Cholesterol Assay Kit according to the manufacturer's instructions.

### Quantification of intracellular cAMP stimulated by forskolin

Cells were pre-incubated with ACi (20 μM) for 1 hour, after which time the cell medium was changed. This was then followed by a 30 min treatment with FSK (20 μM) and ACi (20 μM). After 30 min, adipocytes were lysed with 0.1 M HCL and after dissociation and centrifugation, the cAMP was assayed in the supernatant by the cAMP Direct Immunoassay Kit according to the manufacturer's instructions.

### Quantification of apoE secretion by ELISA

In the apoA-I stimulated apoE experiment, cells were treated with apoA1 dose response (5, 10, 20 μg/ml) for 6, 12 and 24 hours.

In the BFA (5 μg/ml) experiment, cells were pre-incubated with BFA for 12 hours followed by BFA and with or not apoA-I for 6 hours. Culture medium samples were collected and assayed for apoE with The AssayMax Human ApoE ELISA (Assaypro LLC, USA) according to the manufacturer's instructions.

### RNA extraction, reverse transcription and real-time quantitative PCR

Cells from 6 wells (3 × 10^5 ^cells) were extracted with 500 μL of TRIzol™ reagent (Invitrogen, France). Total RNA extraction was performed using the FastRNA^® ^Pro Green Kit (Qbiogene Ltd, France) according to the manufacturer's instructions. Further mRNA purification was then performed using TRIzol^® ^Plus (Invitrogen). cDNA was synthesized using the cDNA Synthesis System (Roche Applied Science) and purified with a QIAquick PCR Purification Kit (QIAGEN, France). Real time qPCR was performed using Taqman probes (ABI, Applied Biosystem Instruments, France) according to the manufacturer's instructions. Gene expression analysis was performed using a LightCycler480 RTqPCR cycler (Roche Applied Science). Analysis was conducted using the Δ-Δcycle threshold (ct) method, which determined fold changes in gene expression relative to a control-treated sample. Each analysis reaction was performed in duplicate, with 6 samples per condition. Gene expression was normalized to glyceraldehyde-3-phosphate dehydrogenase (GAPDH), which was used as an internal reference gene.

### Statistical analysis

Statistical analysis was performed using Microsoft Excel software. Differences were tested for significance (P < 0.001, P < 0.01 and P < 0.05) by the unpaired Student's t-test.

## Results

### ApoA-I increases cholesterol release in mature human adipocytes

**Figure 1:**
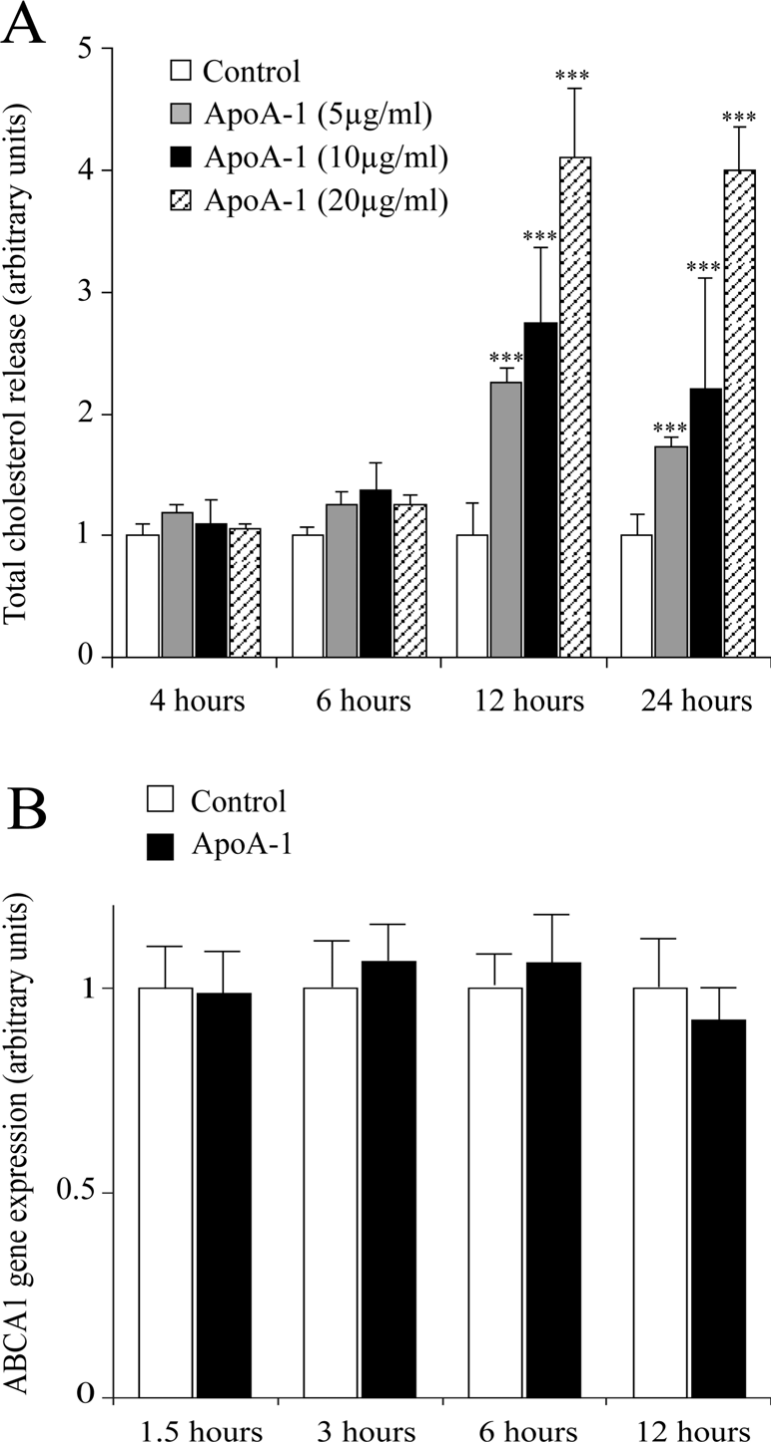
**ApoA-I stimulates total cholesterol release**. **Panel A**: Total cholesterol release was measured in the medium of mature adipocytes treated or not with apoA-I (5, 10 or 20 μg/ml) at 4, 6, 12 and 24 hours by fluorometric dosage. The graph represents the mean ± SD of the results from 4 patients (n = 6 for each condition, for each patient) and all raw data has been normalised *versus *control. ***P < 0.001%, **P < 0.01%, *P < 0.05%, *versus *untreated cells. **Panel B: **ABCA1 gene expression was determined at 1.5, 3, 6 and 12 hours in mature adipocyte cultures, treated or not with apoA-I (10 μg/ml). The graph represents the mean ± SD of the results from one patient (n = 5 for each condition), representative of two experiments on two different patients.

It is widely accepted that apoA-I stimulates the efflux of cholesterol in many cellular models. This has also been demonstrated in murine adipocyte cell lines such as the 3T3-L1 [18], but to date there are no reports that a study being conducted on primary cultures of human adipocytes. Initially, we aimed to investigate the effect of apoA-I. Figure 1 clearly shows that apoA-I increases in a dose dependent manner the release of cholesterol from mature human adipocyte cultures (panel A). We have detected up to a 400% increase after 12 hours, with this level sustained for up to 24 hours. Treatment of the cells with apoA-I does not lead to a simultaneous increase in ABCA1 transcription (panel B).

### ApoA-I induced cholesterol release is cAMP and PKA independent

**Figure 2:**
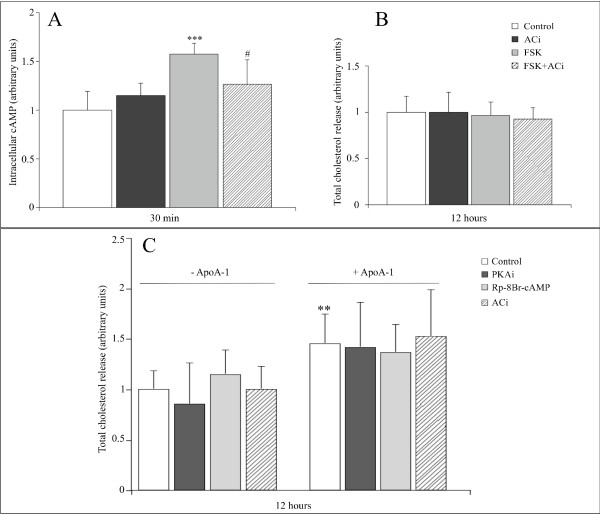
**ApoA-I induced cholesterol release is cAMP and PKA independent**. Depending upon the conditions, mature adipocytes were pre-treated or not for 1 hour with ACi (20 μM), PKAi (40 nM) or rp-8-Br-cAMP (40 μM). Cell medium was removed and cells were either treated or not with ACi or PKAi, rp-8Br-cAMP, FSK (20 μM) and with or without apoA-I (10 μg/ml) for 30 min or 12 hours. **Panel A: **cAMP was quantified in adipocytes, treated or not with ACi and FSK at 30 min, by competitive ELISA. **Panel B: **total cholesterol was measured in the medium of mature adipocytes cultures, treated or not with ACi and FSK at 12 hours by fluorometric assay. **Panel C: **total cholesterol was measured in the medium of mature adipocytes cultures, treated with different inhibitors and with or without apoA-I at 12 hours by fluorometric assay. Graphs represent the mean ± SD of the results from 4 patients (n = 6 for each condition, for each patient) and all raw data has been normalised *versus *control. ***P < 0.001%, **P < 0.01% *versus *control cells and #P < 0.1% *versus *apoA-I treated cells.

Previous studies have shown that apoA-I induces an increase in ABCA1 phosphorylation, as well as an increase in the intracellular levels of cAMP [19], suggesting that the stimulatory effect of apoA-I could be related to cAMP, and thus to PKA [12,13]. However, it would appear that the results may be dependent upon the species and the cellular model studied [20]. Figure 2 shows that treatment of adipocytes with forskolin leads to an increase of intracellular cAMP, after 30 minutes of treatment (panel A). This increase could be prevented by the use of a specific adenylate cyclase inhibitor (panel A). Conversely, the forskolin action does not induce an increase in cholesterol secretion at 12 hours (panel B). This finding was checked at 1, 2, 4 and 6 hours with no visible effect (data not shown). Moreover, the use of specific PKA inhibitors (PKAi and Rp-8Br-cAMP) or adenylate cyclase inhibitor does not lead to a reduction in the activator effect of apoA-I (panel C). It should be noted that even the adipocyte basal release of cholesterol is not modified by PKA or the adenylate cyclase inhibitor.

### Basal cholesterol release but not apoA-I stimulated cholesterol release is inhibited by brefeldin A (BFA)

Numerous studies using different cell types have demonstrated that brefeldin A (BFA) inhibits cholesterol efflux [21,22]. In particular, *Verghese et al*. have demonstrated in the murine adipocyte cell line, 3T3-L1, that the basal efflux of cholesterol is very strongly inhibited by BFA [22].

Given that well established differences between murine and human cells and between cell lines and primary cell cultures we decided to determine the effect of BFA on cholesterol release in mature human adipocytes.

**Figure 3:**
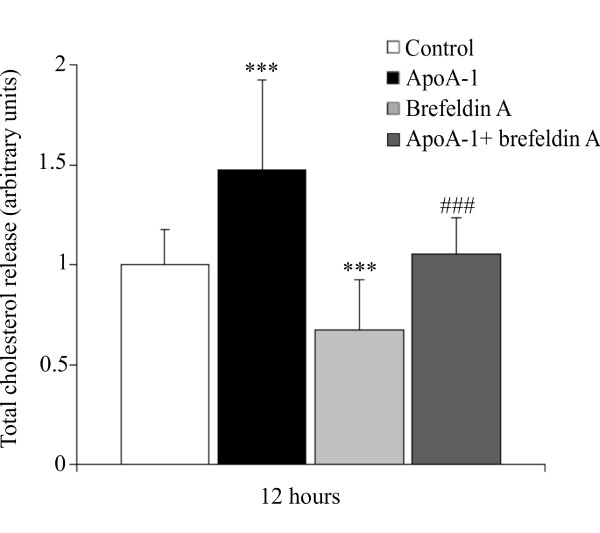
**Brefeldin A inhibits total cholesterol release in mature human adipocytes**. Mature adipocytes were pre-treated with BFA (5 μg/ml) for 12 hours followed by treatment with or without apoA-I (10 μg/ml). Total cholesterol was measured in the medium of mature adipocytes cultures at 12 hours by fluorometric dosage. The graph represents the mean ± SD of the results from 3 patients (n = 6 for each condition, for each patient) and all raw data has been normalised *versus *control. ***P < 0.001% *versus *control cells and ###P < 0.001% *versus *apoA-I.

Figure 3 clearly shows that in mature human adipocytes, cholesterol release is sensitive to BFA. Indeed, following treatment for 12 hours, BFA inhibits approximately 30% of the basal cholesterol release and 35% of the apoA-I stimulated cholesterol release. In fact, the reduction in cholesterol release observed during co-treatment with BFA and apoA-I can be explained by the effect on the basal release. Consequently, BFA has no effect upon the apoA-I stimulated release of cholesterol.

### ApoA-I induced cholesterol release is not linked to cholesterol synthesis

**Figure 4:**
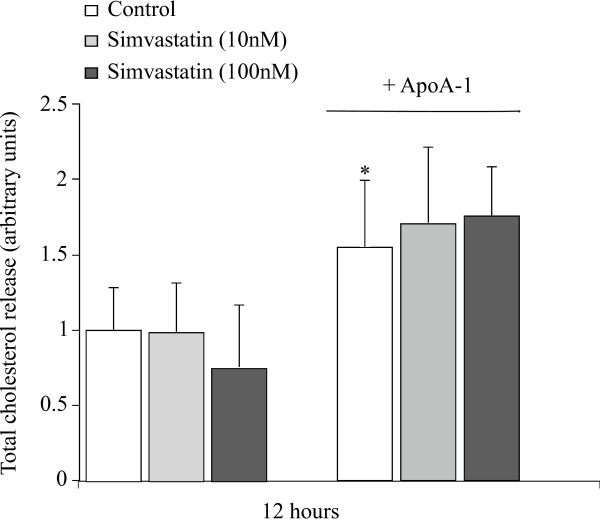
**ApoA-I induced cholesterol release is not inhibited by statins**. Mature adipocytes were pre-treated with simvastatin (10 and 100 nM) for 1 hour followed by treatment with or without apoA-I (10 μg/ml). Total cholesterol was measured in the medium of mature adipocytes cultures at 12 hours by fluorometric dosage. The graph represents the mean ± SD of the results from 3 patients (n = 6 for each condition, for each patient) and all raw data has been normalised *versus *control. *P < 0.05% *versus *control cells.

In order to determine whether the apoA-I induced efflux derives from a regulatory pool of cholesterol and is therefore associated with an increase in HMG-CoA reductase activity, we treated the adipocytes with a statin, which is a known inhibitor of this enzyme. Figure 4 shows that basal cholesterol release is strongly reduced with simvastatin. Nevertheless, this statin has no effect on cholesterol release. Surprisingly, no studies, as far as we are aware, has yet investigated the direct effect of statins on cholesterol release from human adipocytes.

### ApoA-I increases apoE secretion with minimal effect upon apoE gene expression

**Figure 5:**
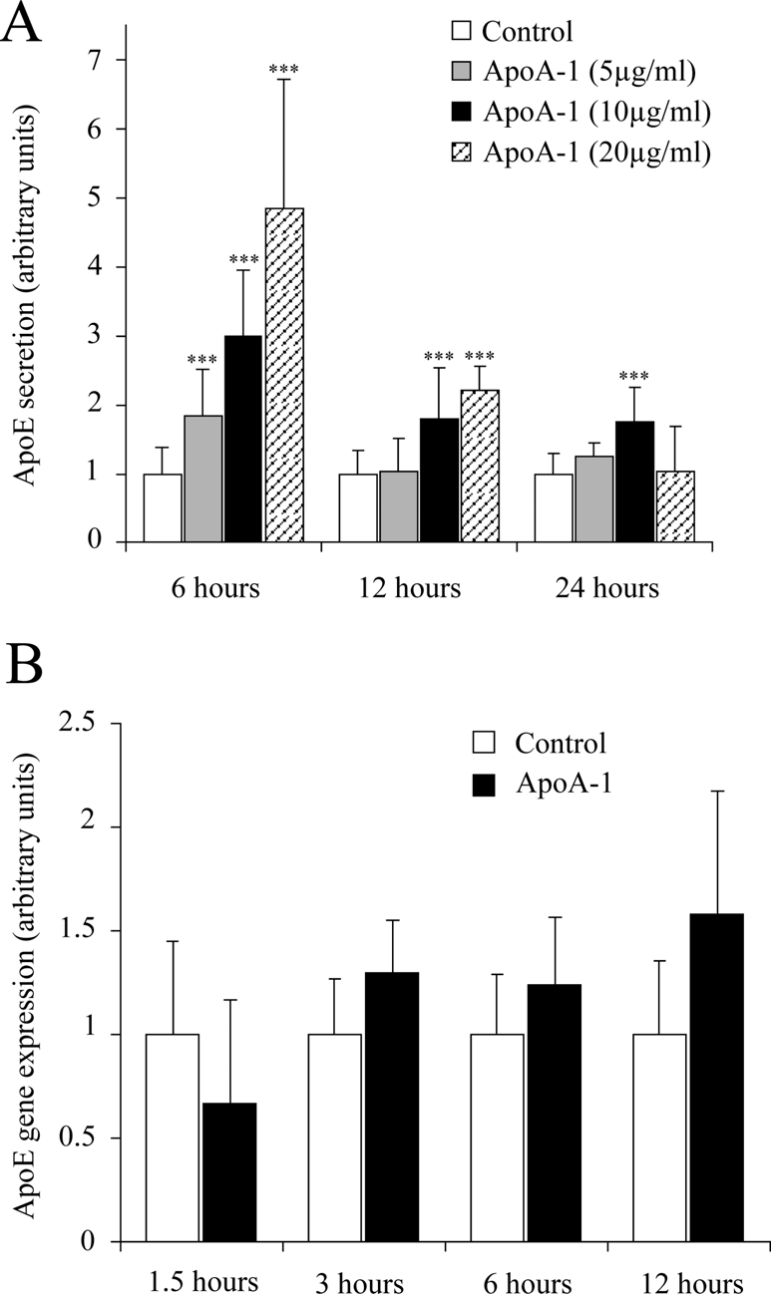
**ApoA-I stimulates apoE secretion in mature human adipocytes**. **Panel A**: ApoE secretion was measured in the medium of mature adipocytes cultures treated or not with apoA-I (5, 10 and 20 μg/ml) at 6, 12 and 24 hours by ELISA. The graph represents the mean ± SD of the results from 4 patients (n = 6 for each condition, for each patient). All raw data has been normalised *versus *control. ***P < 0.001%, *versus *control cells. **Panel B: **ApoE gene expression was determined at 1.5, 3, 6 and 12 hours in mature adipocytes cultures treated or not with apoA-I (10 μg/ml). The graph represents the mean ± SD of the results from one patient (n = 5 for each condition), representative of two experiments on two different patients.

In addition to increasing cholesterol efflux, apoA-I is known to activate apoE secretion, at least in macrophages. Figure 5 shows that a similar effect is also clearly observed in adipocytes, since an increase of 100 to 500% in the amount of apoE is measured in the culture medium at 6 hours, dependent upon the dose of apoA-I used (from 5 to 20 μg/ml). This effect is still measurable up to 24 hours after treatment, although the difference is no longer significant at this time point. Importantly, under these conditions apoA-I does not have a significant impact on apoE gene transcription (panel B).

### Brefeldin A inhibits basal apoE secretion but not apoA-I induced apoE secretion

In order to clarify how apoA-I increased the adipocyte secretion of apoE, we chose to use the Golgi secretion inhibitor, BFA.

**Figure 6:**
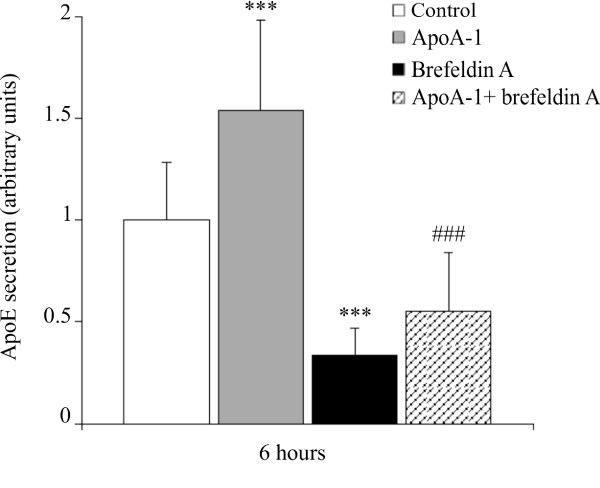
**Brefeldin A inhibits basal apoE secretion but not the apoA-I induced apoE secretion in mature human adipocytes**. Mature adipocytes were pre-treated with BFA (5 μg/ml) for 12 hours and apoE secretion was measured in the medium of matures adipocytes cultures treated or not with apoA-I (10 μg/ml) at 6 hours by ELISA. The graph represents the mean ± SD of the results from 4 patients (n = 6 for each condition, for each patient). all raw data has been normalised *versus *control.***P < 0.001% *versus *control cells, ###P < 0.001% versus apoA-I cells.

Figure 6 illustrates the effect of treating adipocytes with apoA-I, BFA (5 μg/ml), or both together, at 6 hours, corresponding to the apoE secretion peak. Whereas BFA effectively decreases the basal secretion of apoE, the results obtained clearly reveal that BFA does not affect apoA-I induced secretion of apoE.

## Discussion

Numerous studies have shown that apoA-I is able to activate cholesterol efflux as well as the secretion of apoE [4,18,23]. However, most of these studies were conducted on macrophages, rodent cell lines, or on differentiated rabbit adipocytes. These studies have already highlighted fundamental differences depending on the cell type studied [20]. Moreover, given the major differences that exist between these various models and humans; notably in terms of cholesterol HDL levels, the very weak development of atherosclerotic processes, and the absence of CETP in rodents [24], it seemed important that the mechanisms of RCT in human adipose tissue should be investigated.

Until recently, the studies that have been carried out have used labelled cholesterol, enabling the detection of cholesterol following secretion by the cell. This method enables the detection of relatively small effects during cholesterol secretion. The use of the human adipocyte model means that cholesterol "loading" can be avoided, since adipocytes produce and secrete sufficient basal concentrations of cholesterol, with quantification possible *via *a simple fluorometric method. For this reason, we talk about cholesterol release and not cholesterol efflux, because the cells were not previously loaded with exogenous cholesterol.

Our investigation clearly shows that apoA-I effectively increases the release of cholesterol, as well as largely increasing the secretion of apoE by mature adipocytes (figures 1 and 5). These two concomitant effects most probably contribute to the potentiation of the RCT *via *the expansion of HDL2 and HDL1. Indeed, the presence of apoE is crucial to the size increase of the HDLs [6]. It is even more probable, that the presence of HDL1 (HDL-with apoE) has been underestimated until very recently [25]. Indeed, the activation of apoE secretion by apoA-I may not only lead to an optimum increase in the size of the HDLs, but also to an increase in the uptake of these particles by the liver *via *the apoB/E LDLR according to the amount of apoE present.

As far as the regulation of cholesterol efflux within mature adipocytes is concerned, we have highlighted several major points.

First, the effect of apoA-I seems totally independent of the increase in intracellular cAMP, since treatment of the adipocytes with forskolin effectively leads to an increase in the intracellular cAMP, but not to an increase in cholesterol release (figure 2). Moreover, treatment with PKA and adenylate cyclase inhibitors does not lead to a reduction in apoA-I induced cholesterol release (figure 2). This is in line with the results obtained in other cellular models [20] showing that the efflux of cholesterol from human macrophages is independent of cAMP and PKA.

Secondly, the use of BFA inhibits basal cholesterol release from adipocytes (figure 3). This finding concurs with other studies to the effect that BFA partially or completely inhibits basal cholesterol efflux [22]. The fact that BFA disturbs intracellular vesicular trafficking, as well as the dynamics and potentially the intracellular distribution of ABCA1, suggests that BFA impacts the rate of cholesterol efflux through its action on vesicular transport. However, figure 3 clearly shows that BFA does not modify the action of apoA-I on cholesterol since an activator effect persists.

Thirdly, apoA-I does not induce an increase in extracellular cholesterol by the activation of HMG-CoA reductase since the use of specific inhibiting molecules does not block the effect of the apoA-I (figure 4). Nonetheless, it should be mentioned that the statins are highly efficient on adipose cells, because after 24 hours of treatment, the basal or apoA-I induced cholesterol detected in the culture medium strongly decreases (data not shown). Inhibition of HMG-CoA reductase by simvastatin has no effect on the apoA-I induced release of cholesterol (figure 4) suggesting that the increase in cholesterol in the cell medium derives from a cholesterol storage pool not related to a feed-back activation of HMG-CoA reductase. The outcome is identical after treatment with lovastatin (data not shown). However, following 24 h treatment with the statins (data not shown), concomitantly or not with apoA-I, the release of cholesterol into the medium decreases significantly, clearly demonstrating that the statins are active in adipocytes, limit cholesterol formation in the cell, and thus its release into the culture medium. This is definitely an important result in that as far as we know, the role of statins on adipocytes has never been investigated. This suggests that clinical effects of statins are probably due, at least in part, to their action within adipose tissue, particularly in adipocytes.

The overall evidence collected suggests that the increase in apoA-I induced cholesterol efflux is mediated by a particular pathway in human adipocytes. Certain studies clearly demonstrate that PKA plays a key role in the phosphorylation of ABCA1 that leads to its activation [19], whilst BFA disturbs vesicular traffic as well as the intracellular distribution of ABCA1 [26]. However, our results prove that neither the inhibitors of PKA, nor BFA are able to disturb the release of apoA-I induced cholesterol. Moreover, this release is not linked to the transcriptional regulation of the ABCA1 gene, because apoA-I induced efflux does not lead to an increase in ABCA1 gene expression (figure 1). This suggests that the effect of the apoA-I is not directly linked to the activation of ABCA1, or at least, that in adipocyte, the activation of ABCA1 occurs through an undescribed signaling pathway. In addition, treatment with PKC activators and inhibitors does not modify the effect of apoA-I (data not shown), whereas certain studies have suggested the involvement of this pathway [20]. Therefore, this further supports the argument that a particular and probably specific pathway in the adipocyte does exist.

The regulation of apoE secretion by apoA-I, is neither dependent upon an increase in gene transcription, nor upon increased release from the Golgi (figures 5 and 6). It may therefore be assumed that, in macrophage models, apoE is stored mainly in the cytoplasm and/or on the cell surface, with apoA-I enabling secretion of this cytoplasmic pool [4,23]. The same situation also seems to occur in human adipocytes. Moreover, it is noteworthy that inhibition of adenylate cyclase or PKA does not modify the activator effect of apoA-I on apoE secretion (data not shown). This demonstrates that secretion does not require an increase in intracellular cAMP nor a PKA specific activation.

The apoE is highly implicated in the regulation of lipid metabolism, in the expression of certain adipocyte genes, and in the proliferation of pre-adipocytes [7,27,28], with the secretion of apoE being controlled during adipocyte differentiation [29]. We show here that apoA-I strongly stimulates apoE secretion, suggesting that in pathological conditions, such as obesity, the combination of low HDL-c levels (carrying apoA-I) and oxidative stress contributes to a major reduction in adipocyte apoE secretion. This could lead to a reduced apoE protective effect, during the development of adipose tissue [7] and, in particular, during inflammation, thus potentially leading to an increased risk of developing atherosclerosis.

In this regard, we recently demonstrated that mature human adipocytes are able to secrete large quantities of pro-inflammatory molecules such as IL-6 or TNFα [30]. There is therefore increasing evidence to support the view that adipocytes are highly involved in the inflammatory phenomenon associated with the development of obesity.

In addition, this tissue should no longer be considered simply as a passive fatty-acid storage tissue since numerous studies now demonstrate that it acts as a buffer reservoir for circulating cholesterol [18,22]. Finally, the interactions between pro- or anti-inflammatory molecules and cholesterol efflux are now being highlighted [31,32].

## Conclusion

In conclusion, our work highlights the fact that adipose tissue, particularly adipocytes, are key components of the RCT in humans, and that this mechanism is without doubt specifically regulated within these cells. Given that HDL-c levels are reduced in patients that generally have highly developed adipose tissue (obese and diabetic individuals), we may argue that adipose tissue must be regarded as a key contributing factor to the development of cardiovascular diseases in particular atherosclerosis, in these patients.

## Competing interests

The authors declare that they have no competing interests.

## Authors' contributions

RR and MC conceived the study. KB, RM and ET carried out experiments. RGC and CG participated in data collection. LH and FT helped in analysis and interpretation. RR drafted the manuscript. All authors read and approved the final manuscript.
